# Biogenic Design of the Flexible, Resilient, and Hard Mineral Protector in Door Snails

**DOI:** 10.1002/smsc.202500385

**Published:** 2025-09-28

**Authors:** Yuri Kurihara, Taro Yoshimura, Ilian Häggmark, Rei Ueshima, Motoaki Hayama, Takuto Kishimoto, Nozomi Ono, Taige Hao, David Kisailus, Hidetoshi Takahashi, Hiroyuki Fujimoto, Kentaro Uesugi, Masato Hoshino, Yuya Oaki, Takenori Sasaki, Hiroaki Imai

**Affiliations:** ^1^ School of Integrated Design Engineering Graduate School of Science and Technology Keio University Kanagawa 223‐8522 Japan; ^2^ The University Museum The University of Tokyo Tokyo 113‐0033 Japan; ^3^ Graduate School of Science The University of Tokyo Tokyo 113‐0033 Japan; ^4^ Department of Applied Physics KTH Royal Institute of Technology 114 28 Stockholm Sweden; ^5^ Department of Materials Science and Engineering University of California, Irvine California 92697 USA; ^6^ Shimadzu Corporation Kyoto 604‐8511 Japan; ^7^ Japan Synchrotron Radiation Research Institute (JASRI/SPring‐8) Hyogo Japan Hyogo 679‐5198 Japan

**Keywords:** biomimetic materials, hierarchical architectures, mollusk shells, snail evolution, structure–function relationships

## Abstract

Organism design incorporates diverse materials with varying properties, such as hard skeletons of biogenic minerals and soft organic skins. However, achieving a balance of flexibility, resilience, and hardness remains a challenge even for organisms. Door snails have a calcareous door (clausilium) that covers the aperture. The clausilium combines hardness for defense and flexibility for opening and closing. Here, this work focuses on the biogenic design of a clausilium stalk as a unique architecture balancing several properties. This study investigates the stalk, a twisted ribbon with high flexibility and resilience, which is identified as a synapomorphic structure in 22 Clausiliidae species across seven subfamilies and 17 tribes. Internal observations reveal a double‐layered structure: a hard, dense envelope with aragonite rods arranged in the *b* axis and a flexible, low‐density core with randomly packed aragonite nanoparticles and organic matter. The anisotropic hierarchical design seen in nature is surely useful in the development of artificial materials that combine flexibility, resilience, and hardness.

## Graphical abstract

A calcareous door (clausilium) of door snails combines hardness for defense and flexibility for opening and closing. The biogenic design of a clausilium stalk is studied as a unique architecture balancing several properties. Internal observations reveal a double‐layered structure: a hard, dense envelope with aragonite rods and a flexible, low‐density core with randomly packed aragonite nanoparticles and organic matter.

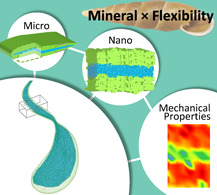

## 1 Introduction

The bodies of living organisms require robustness and resilience to maintain their shape and defense.^[^ ^1^, ^2^, ^3^, ^4^, ^5^ ^]^ Using common elements, organisms exhibit intricate material designs,^[^ ^6^, ^7^, ^8^, ^9^ ^]^ relying on soft and flexible materials such as proteins^[^ ^10^, ^11^, ^12^ ^]^ and polysaccharides,^[^ ^13^, ^14^, ^15^ ^]^ alongside hard and durable materials, such as amorphous silica^[^ ^16^, ^17^, ^18^ ^]^ and crystalline calcium carbonates^[^ ^19^, ^20^, ^21^, ^22^, ^23^, ^24^ ^]^ and phosphates.^[^ ^25^, ^26^, ^27^ ^]^ Organic compounds are integrated into inorganic materials to control their mechanical properties so as to balance flexibility, resilience, durability, and hardness.^[^ ^28^, ^29^, ^30^, ^31^ ^]^

**Figure 1:**
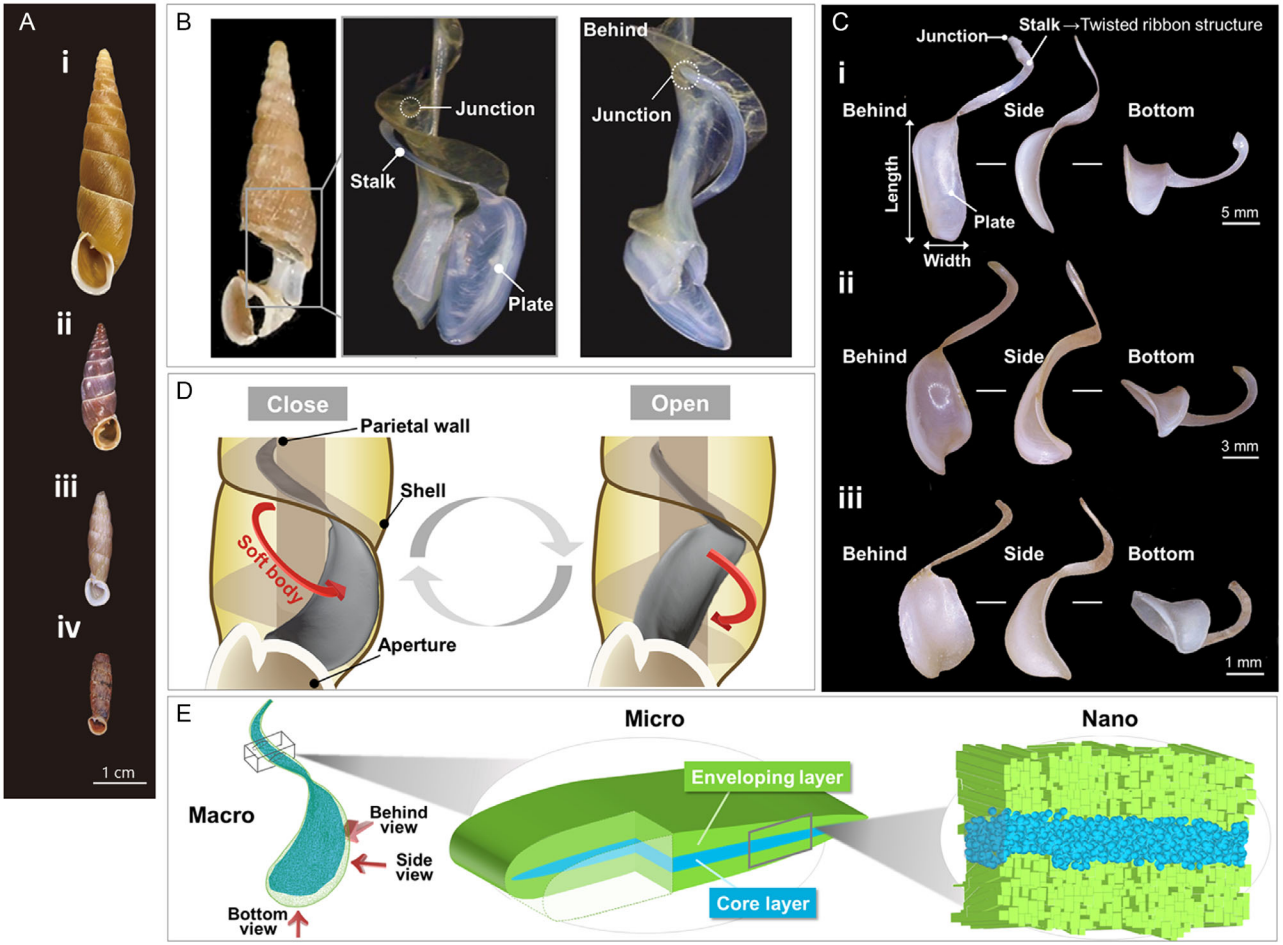
Typical shells and the door (clausilium) of the family Clausiliidae. A) photographs of i) *Megalophaedusa martensi* (Phaedusinae), ii) *Alopia livida* (Alopiinae), iii) *Nenia tridens * (Neniinae), and iv) *Brevinenia richardsi* (Peruiniinae). B) Photographs of the clausilium consisting of a stalk and a plate. The left image shows a door snail with the shell cut out only around the clausilium. The clausilium stalk joins the main shell structure at the position at the back of the right image. C) Photographs of the clausilium from i) *Megalophaedusa martensi*, ii) *Stereophaedusa japonica japonica*, and iii) *Tauphaedusa tau*. D) A schematic illustration of its opening and closing action. E) A schematic illustration of the hierarchical design of a clausilium stalk. The directions of observation in (C) are indicated by red arrows in (E). Three species in (C) have unique plate shapes.

**Figure 2:**
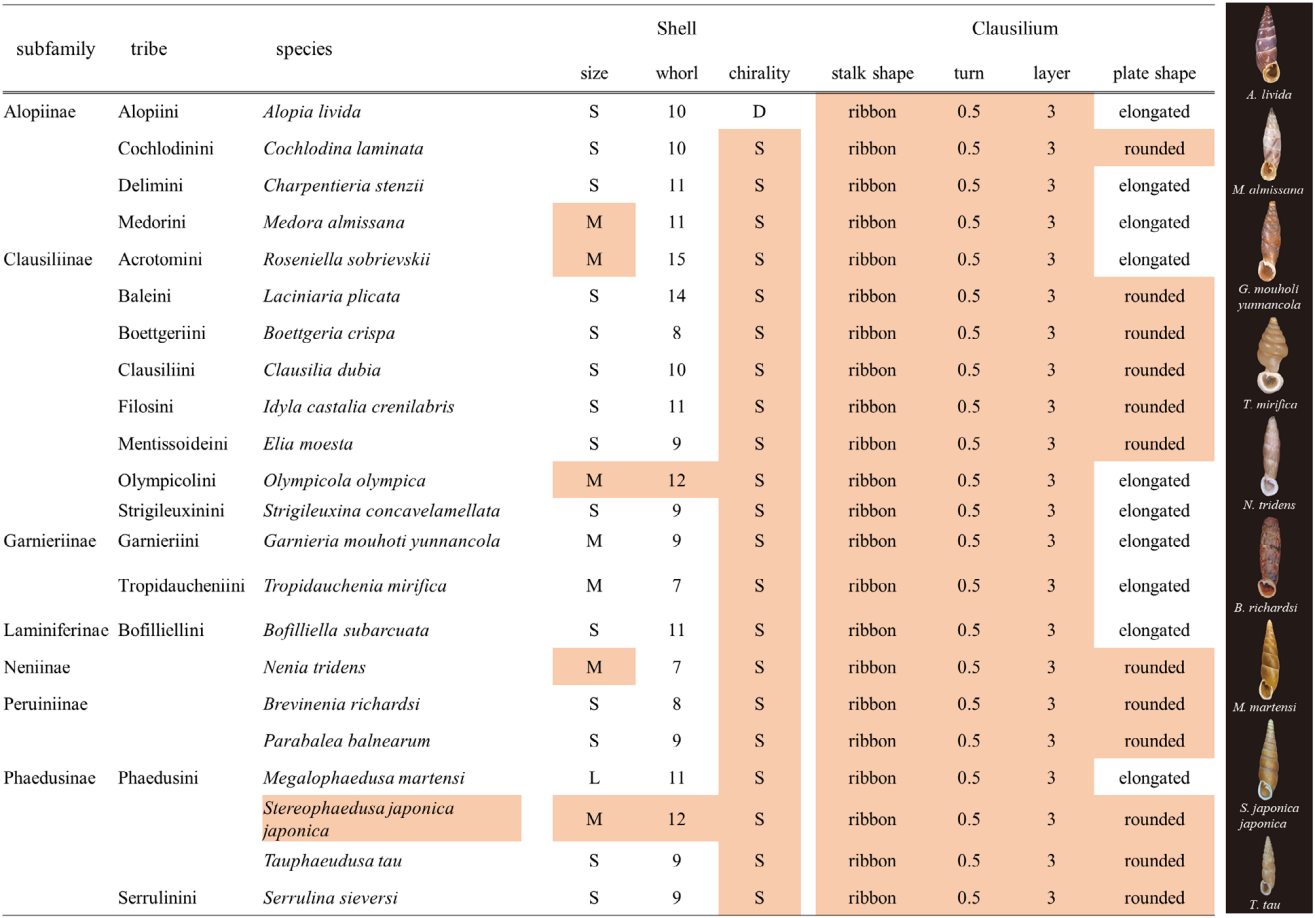
Phylogenetic comparison of shells and clausilia in the family Clausiliidae and photographs of shells. The shell morphologies are based on their respective original descriptions. The phylogenetic classification was constructed with reference to the tribe‐level phylogenetic relationships based on the 28S rRNA sequence.^[^ ^66^ ^]^ The detailed specifications for each abbreviated category are as follows: size—shell length of 40 mm or more (L), those between 40 and 20 mm (M), and those 20 mm or less (S); whorl—the number of body whorls; chirality—the shell coiling of sinistral (S) and dextral (D) snail; stalk shape—the outline of the stalk; turn—the number of turns in the stalk; layer—the number of layers in the microstructure of the transverse section of the stalk (for details, see the next section); plate shape—the plate morphology with width/length ratio <1/2 (elongated: see Figure 1Ci), and ≥1/2 (rounded: see Figure 1Cii,iii). Species sharing common characteristics with the reference *S. japonica japonica*, for which detailed crystallographic analysis was conducted, are highlighted in red. The specimen images on the right correspond to the species as indicated in the table.

Calcium carbonate shells formed by mollusks are among the most complex and diverse biominerals in terms of their morphology,^[^ ^32^, ^33^ ^]^ microstructure,^[^ ^34^, ^35^, ^36^ ^]^ and mechanical properties.^[^ ^37^, ^38^, ^39^, ^40^ ^]^ Generally, the strength design of shells is engineered to withstand external loads from all directions.^[^ ^41^, ^42^ ^]^ However, their structural complexity arises from ecological,^[^ ^37^, ^38^ ^]^ environmental,^[^ ^39^, ^40^ ^]^ and genetic developmental factors.^[^ ^43^, ^44^ ^]^ Since Darwin's era, the intricate shells of snails have fascinated scientists and served as compelling examples of natural selection in evolutionary studies.^[^ ^45^ ^]^ The terrestrial snail family Clausiliidae has a calcareous door‐like plate called a clausilium, which functions as an operculum to close the aperture of the shell (**Figure** **1**). This complex synapomorphic device is flexible and enables the snail to effectively seal its shell. The clausilium is thought to have a specialized design that allows elastic deformation (Movie S1, Supporting Information), which is not seen in other calcium carbonate‐based biomaterials. The door snail originated in early Eocene Europe around 66 million years ago.^[^ ^46^ ^]^ Today, more than 1500 species of the door snail are distributed primarily across Eurasia and South America.^[^ ^47^ ^]^ Extensive biological research regarding the door snail has focused on ecology, phylogeny, and evolution, due to their diverse habitats and species.^[^ ^48^, ^49^, ^50^, ^51^, ^52^ ^]^ Diversities in size, overall outlines, and other microscopic traits of shells were reported for various door snails (**Figures** 1A and **2**).^[^ ^53^ ^]^ However, the biogenic design of the clausilium that provides interesting mechanical properties has not been investigated sufficiently. The clausilium comprises a plate serving as an operculum and a stalk connecting to the inner wall of the shell (Figure 1B–D). Especially, the stalk achieves a rare combination of hardness and durability for defense against predators and flexibility and resilience for opening and closing action. The key factors for the balancing of several properties of the unique biogenic calcareous materials have been hidden in the shell.

This study aims to elucidate the biogenic design of the clausilium, which provides remarkable, flexible, and resilient properties of calcareous parts. As shown in Figure 2 and Table S1, Supporting Information, we systematically examined the hierarchical architecture, including the macroscopic shape, microscopic internal structure, and nanoscopic crystalline features of 22 species of door snails, including seven subfamilies and 17 tribes. The morphology of the adult shells examined in this study exhibited sizes ranging from ≈10 to 50 mm, with the number of body whorls varying from 6 to 12. Furthermore, the complex traits of the shell aperture demonstrate taxonomically significant diversity, and traditional phylogenetic classifications have been based on combinations of these characteristics.^[^ ^54^ ^]^ On the other hand, in the flexible stalk part of the clausilium, no morphological differences were observed in all species, such as a twisted ribbon structure (Figure 1C). This suggests that the shape of the stalk has been optimized for a specific mechanical function during the course of evolution. Additionally, microstructural analysis using synchrotron micro‐X‐ray computed tomography (X‐ray CT) and nanoindentation unveiled that the clausilium is composed of a continuous double‐layered structure consisting of a relatively soft core and a rigid envelope (Figure 1E). Furthermore, electron microscopy, electron backscattered diffraction (EBSD), and compositional analysis shed light on the nanoscale crystallographic features. Unraveling the hierarchical material design not only provides crucial insights into how unique properties are acquired by snails but also serves as a blueprint for developing novel functional materials. The unique combination of mechanical properties and hierarchical structural features found in the clausilium may offer valuable insights for biomimetic materials design. Understanding how hardness, flexibility, and resilience are integrated within this natural system could potentially guide the development of synthetic composites with multifunctional capabilities, contributing to the advancement of biomimetic approaches in materials science.

## 2 Results

### 2.1 Macroscopic Characteristics of the Stalk

In general, the macroscopic morphology of the stalk is associated with the opening and closing action of the door. As shown in Figure 2, the complex shell morphology of door snails exhibits diversification across various characters within the family. On the other hand, we propose that this uniformity is an evolutionary conservatism that enables the specialized function of significant elastic deformation in the clausilium.

**Figure 3:**
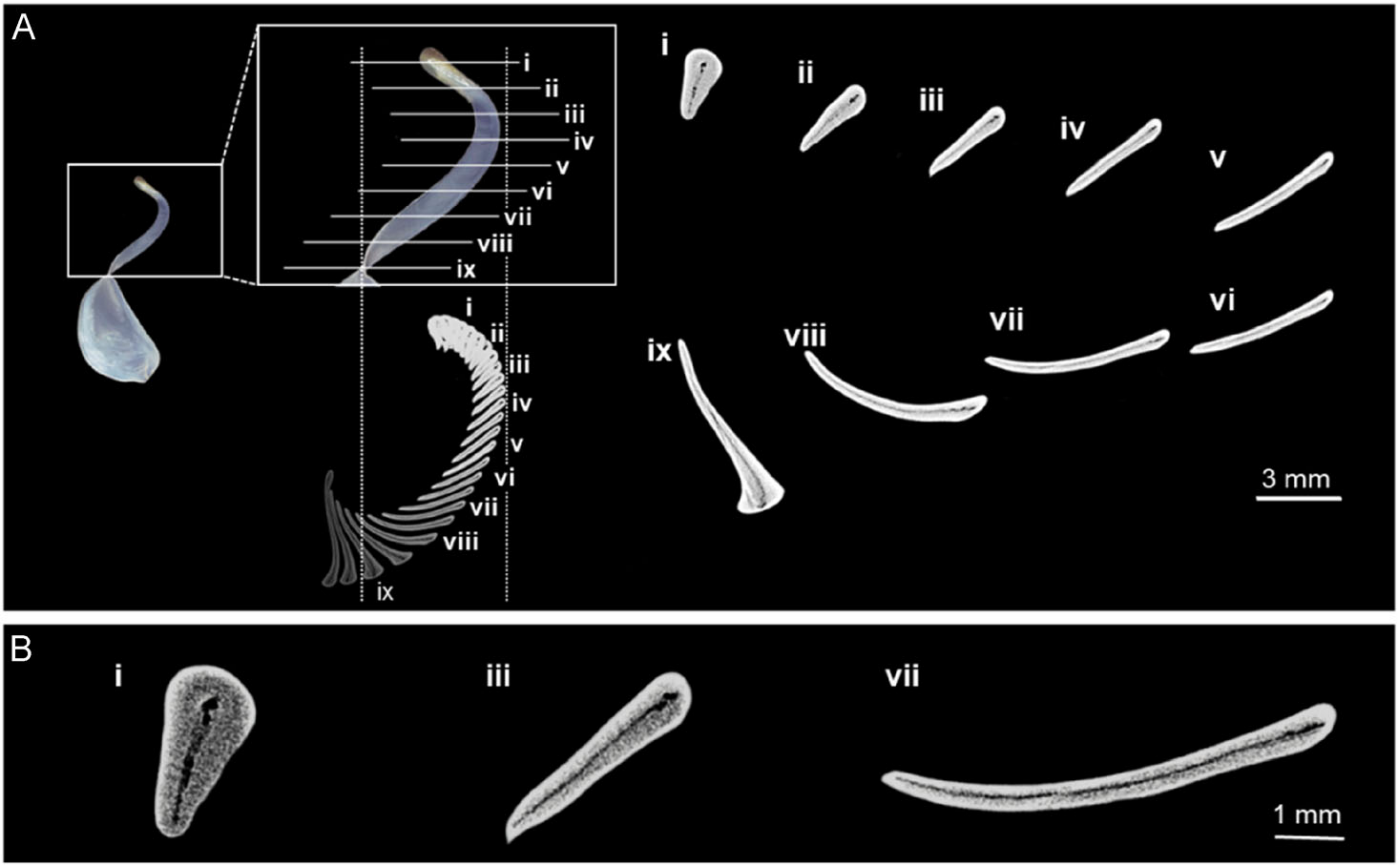
Photo and X‐ray CT images of a clausilium stalk. A) transverse section images at multiple locations i–ix) in a stalk. i) Closest to the junction where it joins the main shell structure. B) Magnified views of i), iii), and vii).

Basically, the shape of the plates depends on the shell aperture. In contrast to the diversity in the morphology of their shells and plates (Figures 1A,C, and 2), the stalks have a similar ribbon‐like form (**Figures** 1, 2–**3**). Although the size depends on that of the shell, the ratios of the maximum width, maximum thickness, and length of the ribbons are almost the same. Moreover, the twisted morphology and variation of the transverse section of the stalks are also characteristic for the clausilium. The ribbon of the stalk is twisted about 180° from the junction connecting the parietal wall to the region connecting with the plate (Figures 1C and 3A). The twisted ribbon of the stalk supporting the flexibility of the clausilium exhibits a near‐triangular cross section close to the junction of the main shell structure and gradually transitioning to a flatter cross section near the plate. This transition is attributed to the need to maintain strength and rigidity near the junction, hence, the adoption of a circular or less flat cross section. This ensures structural stability and minimizes deformation in response to external forces or loads.^[^ ^55^ ^]^ Conversely, the flattening of the cross section toward the plate enhances the flexibility and deformability of the structure, facilitating a sequential propagation of deformation from the junction to the plate in response to external forces. Moreover, the flattened cross section effectively diffuses stress and evenly distributes deformation. Thus, the spring‐like structure optimizes its structural mechanics by featuring a circular or near‐less flat cross section at the junction, transitioning to a flatter cross section toward the plate, thereby enhancing stability and efficiency in deformation management.

**Figure 4:**
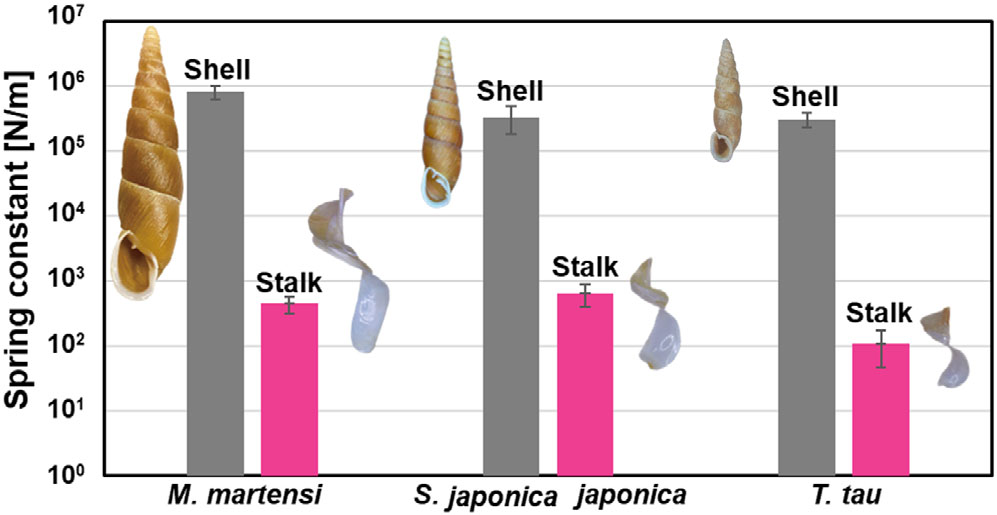
Spring constants of the stalk and shell of three species: *M. martensi* (shell, *N* = 9; stalk, *N* = 8), *S. japonica* *japonica* (shell, *N* = 10; stalk, *N* = 9), and *T. tau* (shell, *N* = 11; stalk, *N* = 6), where *N* = the number of specimens tested per species. The error bars represent the standard deviation.

**Figure** **4** shows the spring constants of the clausilium apparatus and shells of three species of door snails with different sizes: *Megalophaedusa martensi*, *Stereophaedusa japonica japonica*, and *Tauphaedusa tau*. A compression test was used to evaluate the spring constants of the clausilium, where the measurement samples were prepared to align the test loading direction with the actual load direction in nature. The spring constants of clausilium were determined to be 10^2^–10^3^ N m^−1^, whereas those of the shells were 10^5^–10^6^ N m^−1^. The low values for the clausilium are attributable to the flexibility of the stalk. The clausilium visually recovered its original shape after deformation under quasistatic loading, indicating that the stalk exhibits flexibility and apparent resilience. As shown in Movie S1, Supporting Information, the clausilium repeatedly deforms and returns to its functional shape during natural opening and closing motions. Importantly, such reversible deformation occurs thousands of times throughout the lifetime of the door snail, which is known to live for over a decade. The ability of the clausilium to repeatedly deform without structural failure over such a long period suggests that the structure is naturally adapted to withstand repeated elastic deformation. Low‐cycle bending tests and finite element analysis (FEA) simulations will serve to corroborate the main findings of this study. However, the clausilium itself is very small and highly sensitive. Thus, the experimental setup and operation are difficult, making it challenging to obtain accurate data. Moreover, its intricate hierarchical structure makes it extremely difficult to construct a computational model that could provide reliable simulation results. A more comprehensive mechanical or computational understanding, which involves the mechanical approach, will be pursued in future investigations.

In this study, we conducted a comprehensive phylogenetic comparison of the clausilium, and it is noteworthy that both the morphological and microstructural features are uniform. Scanning electron microscope (SEM) observations of the clausilium cross sections revealed that all 22 species examined in this study are composed of double‐layered structures (Figure S1, Supporting Information), which are described in more detail in the following section. Therefore, in the clausilium of door snails, both macroscopic and microstructural features are uniform. For this reason, our detailed investigation focused on a common species—*S. japonica japonica*—which provides a manageable size for analysis.

### 2.2 Microscopic Internal Characteristics of the Stalk

**Figure 5:**
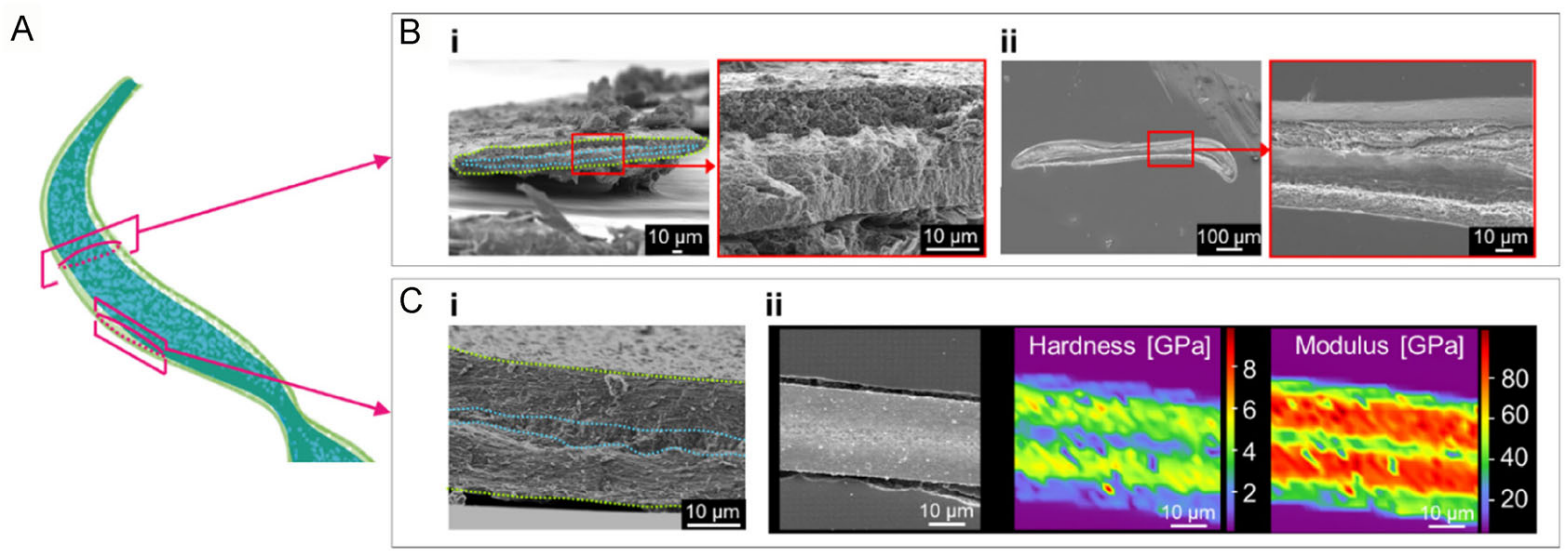
Microscale characteristics in the internal structure of the stalk. A) A schematic illustration based on X‐ray CT images for the double‐layered structure of a clausilium. B) SEM micrographs of a transverse section of the stalk 1) before and 2) after dissolution of the calcium carbonate with EDTA treatment. The blue and green dotted lines suggest the core region and the enveloping layer, respectively. C) 1) SEM micrographs and maps of the hardness and 2) Young's modulus of a longitudinal section of the stalk.

Microscopic structural design features play a significant role in controlling the mechanical properties of biogenic materials.^[^ ^8^ ^]^ The internal structures of the clausilium were precisely analyzed by X‐ray CT and SEM and their resulting local mechanical performance by nanoindentation. As shown in Figure 3B, a thin, low‐density region was found in the transverse section of the clausilium stalk. **Figure** **5** shows SEM micrographs of the transverse and longitudinal sections of a stalk. Evaluation of the transverse section of the stalk (Figure 5B) highlights a double‐layered structure with two types of microstructures: 1) an enveloping layer (5–10 μm thick), consisting of rods, and 2) an inner core (5–10 μm thick), containing nanoparticles. As mentioned above, the double‐layered structures are common in the stalk of the other 19 species (Figure S1, Supporting Information) and are also observed in the plates (Figure S2, Supporting Information). The phases of the rods and nanoparticles were assigned as aragonite‐type calcium carbonate by Raman spectroscopy (Figure S3, Supporting Information). Energy‐dispersive X‐ray spectroscopy (EDX) revealed that the approximate organic contents of the enveloping layers and core are 3 and 30 wt%, respectively (Figure S4A and B, Supporting Information), while thermal gravimetric analysis (TGA) shows that the entire clausilium contains ≈6 wt% organic matter (Figure S4C, Supporting Information). The residual organic matter after the dissolution of calcium carbonate with ethylenediaminetetraacetic acid (EDTA) was analyzed by Fourier‐transform infrared spectroscopy (FTIR) and a staining method (Figures S5 and S6, Supporting Information). Staining with calcofluor white suggested that the residual organic matter is mainly composed of chitin, chitosan, or other kinds of polysaccharides, which are commonly found in many biogenic materials, including mollusk shells that help guide microstructural features.^[^ ^56^ ^]^ Additionally, the presence of a specific absorption band indicated that chitin‐based organic polymer is mainly contained in the core. Although FTIR and calcofluor staining alone cannot provide definitive identification, our results are consistent with previous studies showing that chitin is a major organic component in molluscan shells and other calcium carbonate‐based biomaterials. We therefore present the organic matrix as chitin‐based.^[^ ^6^, ^8^, ^22^, ^35^ ^]^

The mechanical properties of the cross sections of the stalk were evaluated by nanoindentation. As shown in Figure 5C, the hardness (4.3 ± 0.8 GPa) and Young's modulus (80 ± 9 GPa) of the enveloping layer were higher than those of the shells (hardness: 3.8 ± 1.9 GPa; Young's modulus: 74 ± 23 GPa),^[^ ^57^, ^58^ ^]^ while the core layer had a lower hardness (1.9 ± 0.7 GPa) and Young's modulus (41 ± 9 GPa). Notably, the enveloping layer exhibited greater hardness than not only the door snail shell but also other crossed‐lamellar and nacre structures, which are well known for their high hardness and durability (Figure S7, Supporting Information).^[^ ^8^, ^37^, ^59^, ^60^ ^]^ The fact suggests that the majority of the internal structure of the clausilium contributes to the durability, matching that of the durable shell (Figure S8, Supporting Information).

Finally, we determined that the stalk and plate of the clausilium are composed of a double‐layered structure that consists of a mineral‐rich, relatively hard envelope and an organic‐rich, relatively flexible core. Such a double‐layered structure is also found in bovine horns and chiton teeth that require a balance of hardness, durability, and resilience. Bovine horns exhibit an elongated and gently curved morphology and chiton teeth take the form of thin, curved plates. These characteristic macroscale shapes are adapted to their respective mechanical functions. Furthermore, in both cases, the internal structure consists of a relatively hard, mineralized outer layer and a softer, more compliant inner core.^[^ ^61^, ^62^ ^]^ This combination of morphology and internal structure enables these biological systems to achieve the specific mechanical properties. Similar structural motifs are observed in traditional Japanese swords (samurai swords) (Figure S9, Supporting Information).^[^ ^63^ ^]^ By encapsulating a flexible core with a hard, outer layer, the overall durability and impact resistance of the sword are improved. Similarly, in the clausilium, the overall flexibility and suggested resilience are markedly different from those of the shell, even though the hardness and durability of the surface enveloping layer are comparable to those of the shell, possibly due to this double‐layered structure.

### 2.3 Crystallographic Characteristics of the Stalk

**Figure 6:**
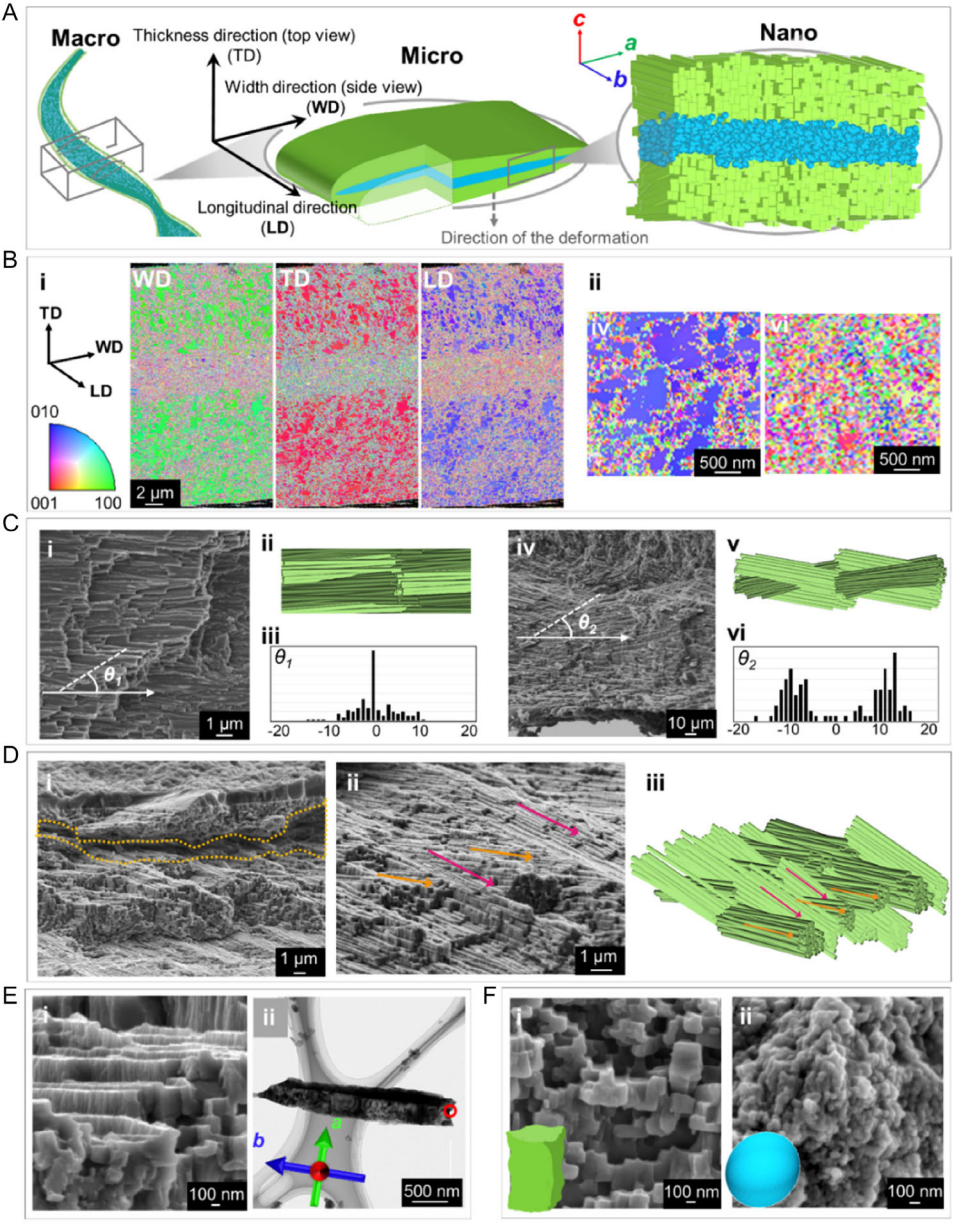
Crystallographic characteristics of the structure of the stalk. A) Schematic outline of the structural design at macro, micro, and nanoscales. B) Inverse pole figure maps of EBSD for three directions (thickness direction (TD), width direction (WD), and longtudinal direction (LD)) on the transverse section of the stalk (i) and (left) the magnified figures of enveloping and (right) core layers; C) characteristics of the rod arrangements in the enveloping layer with SEM, schematic images, and azimuth distribution of rods. i–iii) The results of top view and iv–vi) side view. $\theta_{1}$ represents the angle between the extension direction of the rod and the longitudinal direction of the stalk in the top view. $\theta_{2}$ represents the angle between the extension direction of the rod and the surface of the stalk in the side view. The vertical axis of the azimuth distribution graph indicates the number of rods; D) characteristics of the enveloping layer. i,ii) SEM images of bird's eye view and the schematic image of interwoven bundles structure in the enveloping layer; E) characteristics of crystalline rods in the enveloping layer. i) SEM image and ii) TEM. F) SEM images of i) nanoblocks in the enveloping layer and ii) nanoparticles in the core layer.

The crystallographic characteristics (i.e., shape, size, and orientation) of the stalk were investigated using electron microscopy (**Figure** **6**). Schematic outlines of the structural design at macro, micro, and nanoscales are shown in Figure 6A. According to inverse pole figure maps of EBSD that are almost the same color for three directions (width direction, thickness direction, and longitudinal direction) on a transverse cross section of the stalk (Figure 6B), aragonite crystals were found to be highly oriented in the enveloping layer. Since the (010) plane was exposed in the cross section (Figure 6B–LD), the *b* axis was deduced to be aligned parallel to the longitudinal direction of the stalk. Moreover, the EBSD images for other sides (Figure 6B–WD, TD) indicate that the *a* and *c* axes are also oriented in the width and thickness directions, respectively.

In the enveloping layer, crystalline rods several hundred nanometer thick and several micrometer long were observed to be mainly aligned parallel to the longitudinal direction of the stalk (Figure 6C). Although aligned rectangular blocks were found in the rods (Figure 6E), the spot pattern of selected area electron diffraction (SAED) (Figure S10, Supporting Information) and the EBSD image (Figure 6B(ii)) indicates that the rods are nearly single crystalline elongated in the *b* axis (Figure 6E(ii) and S8B). This suggests that the blocky units are arranged in the same crystallographic direction, like a single crystal (Figure S11, Supporting Information). The lattice orientation of the aragonite crystals is consistent with the overall direction of the stalk, as mentioned above.

Whereas the *b* axis of aragonite crystals is basically aligned parallel to the longitudinal direction of the stalk, we found that the alignment of the rods is modulated in the enveloping layer (Figure 6C,D). In the thickness direction, the rods were arranged in two directions tilted at +10° and −10° from parallel to the wide surface of the stalk. However, the rods are almost parallel to the longitudinal direction in the width direction. As a consequence, an interwoven structure with many voids (Figure 6D(i)) is deduced to be constructed with the aragonite rods.

The core consists of small grains ≈80 nm in diameter (Figure 6F(ii)). The mosaic structure on the EBSD map indicates that the crystallographic direction of the tiny units is random. TEM–EDX results for the core revealed that organic matter fills the gaps between the grains. The flexibility and durability of the core are associated with a mosaic structure consisting of nanograins with the presence of a large amount of organic matter. This was recently observed in a calcium phosphate‐based system, demonstrating interpenetrating bicontinuous organic–inorganic phases that provided a flexible response under quasistatic conditions.^[^ ^64^ ^]^

## 3 Discussion

In this work, we revealed the hierarchical design of the stalk of the clausilium as a specific balancing of the hardness, durability, flexibility, and resilience. Macroscopically, the stalk is a species‐independent twisted ribbon with high flexibility. This macroscopic feature of the stalk manifests a form that exhibits remarkably high evolutionary conservatism as compared to other shell morphologies, suggesting a close association with function.

A macroscopic ribbon, which has an anisotropic flattened morphology, is generally suitable for deformation in the thickness direction. This anisotropic form is adopted as the stalk of the door in the family Clausiliidae. However, the macroscopic morphology is not sufficient for obtaining hardness, durability, and resilience.

Microscopic observations revealed that the stalk has a double‐layered structure similar to that of samurai swords, in which a hard skin and a flexible core combine to provide both strength and toughness. Since the stalk is key to the opening and closing action of the door plate, the relatively soft core that is covered by the relatively hard enveloping layer is required to balance flexibility, hardness, and durability. The core of the stalk and the plate of the clausilium is produced by the random packing of aragonite nanoparticles with a large amount of chitosan‐based polymer. The combination of the inorganic nanoparticles and the organic matter is utilized as a soft layer to prevent brittle fractures.^[^ ^8^, ^59^, ^60^ ^]^

Our study further revealed that the outer hard layer features an interwoven bundled structure of rod‐like biominerals, which maintains hardness while preventing breakage during deformation. The highly oriented aragonite rods form a relatively hard enveloping layer with a small amount of organic matter. The longitudinal direction of the rods is almost parallel to the longitudinal direction of the stalk, and rods are slightly crossed in a certain direction. In general, hard mollusk shells commonly comprise crossed lamellar consisting of aragonite rods.^[^ ^59^, ^65^ ^]^ Interestingly, we observed an anisotropic interwoven bundled structure with many cavities, which help relax the stress from deformation. The specific bundled structure is considered to potentially contribute to the stalk's resilience.

In addition to the microscale double‐layered structure, these nanoscale hierarchical designs contribute to the clausilium's ability to simultaneously exhibit hardness and flexibility.

By elucidating these structural and mechanical features, our work provides valuable insights that may inspire the design of synthetic materials balancing hardness with flexibility. Such bioinspired strategies could contribute to developing advanced composites that require both mechanical strength and controlled deformability.

Further studies are needed to explore the practical application and scalability of these design principles.

## 4 Conclusion

In summary, this study reveals that hierarchical structural designs, ranging from the nanoscale to the macroscale, play a crucial role in imparting the flexibility, durability, and hardness essential for the function of the clausilium. The evolutionarily conservative macroscopic form, at the joint point with the main structure of the shell, possesses a structure that ensures durability. The twisted ribbon structure contributes to the flexibility of the stalk, while the microinternal structure provides hardness, durability, and flexibility. By incorporating a flexible core layer rich in organic material that absorbs load, within a very hard enveloping layer, the clausilium achieves both durability and flexibility while maintaining its hardness. Furthermore, the nanoscale structures that introduce differences in the properties of these two layers set it apart from other shells, ensuring that deformation is not impeded.

## 5 Experimental Section

5.1

5.1.1

##### Materials

Specimens of door snails were collected from each location listed in Table S1, Supporting Information. For species from Eurasia and South America, preserved specimens collected before 1993 in The University Museum, The University of Tokyo (UMUT) were utilized. The specimens used for mechanical properties testing were obtained by removing the soft body from the shell immediately before the experiments. All samples used in the experiments were registered and stored at UMUT.

##### X‐Ray Computed Tomography

Propagation‐based phase‐contrast imaging was performed at beamline BL20B2 at the SPring‐8 Synchrotron in Hyogo, Japan (Proposal 2022A1565). The recently installed double multilayer monochromator was used to achieve a quasimonochromatic (ΔE/E ≈ 4.2%) X‐ray beam with high flux at 40 keV. A 0.3 mm Cu filter removed any low‐energy photons. A rotating piece of bamboo was used as a diffuser to average out fluctuations on a few ms scale. Experiments were performed in hutch 3, resulting in a source‐object distance (*r*1) of ≈207 m. The object‐detector distance (*r*2) was varied between 0 m and 1.3 m. The propagation distance (*z*), which is equal to *r*2, was chosen based on a criterion that achieve the best contrast at a certain frequency, *z* = 1/(2*λf*2). This formula assumes a phase object but gives a rough estimate of the true value. 1/*f* was set according to the point spread function (PSF) of the detector. The PSF was measured to ≈9 μm with a JIMA RT RC‐05 test target. The detector consisted of a single‐crystal scintillator (200 μm GAGG(Ce)), a lens unit, and a Hamamatsu ORCA Lightning sCMOS camera (C14120‐20 P). The camera had a 4608 × 2592 sensor with 5.5 μm pixels and a 16‐bit ADC. The lens unit (two *f* = 105 mm lenses) resulted in an effective pixel size of 5.64 μm (at *r*2 = 0). At 1.3 m, the slight divergence in the X‐ray beam resulted in magnification and thus an effective pixel size of 5.59 μm. An exposure time of 40 ms per image was used. Images were acquired with Hamamatsu HiPic and custom software at the BL20B2 beamline. Tomographies were performed over 180° with 0.1° steps resulting in 1800 projections. The total scan time was <3 min. The dose rate was ≈3.0 Gy/s. The transmission through the sample holder (tube filled with water) at the thickest point was between 50% and 60%.

##### Measurement of Spring Constants

In this study, we used the spring constant as an indicator of flexibility. The spring constants for both the stalk and the shell were measured through different methods.

For the shell, its spring constant was calculated based on the relationship between deformation magnitude and applied force when compressed on a material testing machine (Instron 5944). The shell was fixed at the center of the material testing machine's stage, its mouth facing the bottom. The stage was advanced at a speed of 0.04 mm min^−1^ to compress the shell, continuing until the load's peak was exceeded. Then, a stress–strain diagram was plotted with stress on the vertical axis and strain on the horizontal. Then, the slope of the linear region was identified as the spring constant.

Additionally, the spring constant of the stalk was evaluated. The stalk was extracted from the shell and fixed with the plate part of the stalk facing horizontally upward. The plate was pushed with a needle fixed on a load cell LVS‐50GA, Kyowa Electronic Instruments). The load cell was placed on a piezo stage (6B079‐1, NC3301‐C or PS1L60‐400U‐S and PSVL60‐400U‐S, NCM6201S, THK Precision), which was displaced with a triangular wave motion according to a function generator (WF1974, NF). The stage was moved at 0.1 Hz with a displacement of 100 μm. In the experimental setup, we used a microscope (VH‐5000, Keyence) with a long focal length (VH‐Z50L, Keyence) to monitor the pushing point of the stalk from a topside view. The pushing point was adjusted by a manual optical stage. The spring constant was obtained by plotting the applied displacement versus the force measured by the load cell and calculating the slope by the least‐squares method.

These tests were conducted on 10 samples each for both the stalk and shell across the three species of the door snails, *M. martensi*, *S. japonica japonica*, and *T. tau.* The results were compared based on the average values from these 10 data points.

##### Microscopic Observation

Observations of the shell and clausilium apparatus appearance were made by digital microscopy (DVM6, Leica) and depth compositing was used to acquire images.

The fracture surface and cross section of the clausilium apparatus were examined using field‐emission SEM (JSM‐7600 F, JEOL and Merlin Compact, ZEISS). The cross sections of the clausilium were prepared by polishing after the clausilium apparatus was embedded in epoxy resin, which were etched with a 0.1 wt% ethylene diamine tetraacetate solution to remove damaged topmost layers and then coated with osmium for SEM observation. Fracture surfaces were observed after samples were adhered to the substrate with carbon tape and coated with osmium.

The rods inside a clausilium apparatus were examined using transmission electron microscopy (TEM; Tecnai G2, FEI). Samples were prepared by dispersing crushed clausilium apparatus in ethanol. The dispersion was dropped onto a TEM grid for observation, and SAED pattern were obtained. In addition, samples were prepared by focused ion beam (FIB)–SEM (Quanta 3D FEG, FEI) and examined using TEM–EDX (Tecnai Osirus, FEI).

##### Electron Backscatter Diffraction

EBSD measurements were carried out, after coating with osmium, on a JEOL JSM‐70 001 F field‐emission SEM equipped with an Oxford EBSD detector. Information obtained from EBSD measurements was presented as color‐coded crystal‐orientation maps with corresponding pole figures. Sections were embedded in epoxy resin and polished with colloidal silica for nanoindentation.

##### Nanoindentation Tests

Nanoindentation was performed using a nanoindenter (iMicro, KLA) equipped with a high‐resolution InForce 50 actuator and diamond cube corner tips with a radius of ≈50 nm. Prior to testing, the nanoindenter was calibrated using a standard fused silica sample following the manufacturer's protocol for tip area function and force–displacement calibration. The measurements were conducted under the assumption of a Poisson's ratio of 0.3 for the analyzed material. Tests were conducted at 1050 points with a map size of 70 × 60 μm using the NanoBlitz 3D Rapid Mechanical Property Mapping method based on ISO 14 577. Sections were embedded in epoxy resin and polished with colloidal silica for nanoindentation. The target load was set to 1 mN, and mapping images were obtained by testing at 2 μm intervals.

##### Thermogravimetric Analysis

Clausiliums were cleaned in an ultrasonic bath and ground into a powder. The clausilium powders were then tested with TGA and differential scanning calorimetry (DSC) performed on a TGA/DSC 3 + Mettler Toledo under flowing argon from 25 to 800 °C at a heating rate of 1 °C min^−1^.

## Supporting Information

Supporting Information is available from the Wiley Online Library or from the author.

## Conflict of Interest

The authors declare no conflict of interest.

## Supporting information

Supplementary Material

## Data Availability

The authors declare that the data supporting the findings of this study are available within the paper and its Supplementary Information file. Raw data files are available from the corresponding author upon reasonable request.

## References

[1] P. Y. Chen , J. McKittrick , M. A. Meyers , Prog. Mater. Sci. 2012, 57, 1492.

[2] L. Bergström , E. V. Sturm née Rosseeva , G. Salazar‐Alvarez , H. Cölfen , Acc. Chem. Res. 2015, 48, 1391. 25938915 10.1021/ar500440b

[3] S. E. Naleway , J. R. A. Taylor , M. M. Porter , M. A. Meyers , J. McKittrick , Mater. Sci. Eng. C 2016, 59, 1143. 10.1016/j.msec.2015.10.033 26652472

[4] Y. Chen , Y. Feng , J. G. Deveaux , M. A. Masoud , F. S. Chandra , H. Chen , D. Zhang , L. Feng 2019, 9, 68.

[5] Z. Jia , Z. Deng , L. Li , Adv. Mater. 2022, 34, 2106259. 10.1002/adma.202106259 35085421

[6] U. G. K. Wegst , H. Bai , E. Saiz , A. P. Tomsia , R. O. Ritchie , Nat. Mater. 2015, 14, 23. 25344782 10.1038/nmat4089

[7] Q. Zhang , X. Yang , P. Li , G. Huang , S. Feng , C. Shen , B. Han , X. Zhang , F. Jin , F. Xu , T. J. Lu , Prog. Mater. Sci. 2015, 74, 332.

[8] W. Huang , D. Restrepo , J. Y. Jung , F. Y. Su , Z. Liu , R. O. Ritchie , J. McKittrick , P. Zavattieri , D. Kisailus , Adv. Mater. 2019, 31, 1901561. 10.1002/adma.201901561 31268207

[9] S. H. Kim , M.‐R. Ki , Y. Han , S. P. Pack , Int. J. Mol. Sci. 2024, 25, 6147.38892335

[10] B. Wang , W. Yang , J. McKittrick , M. A. Meyers , Prog. Mater. Sci. 2016, 76, 229.

[11] I. Kellersztein , S. R. Cohen , B. Bar‐On , H. D. Wagner , Acta Biomater. 2019, 94, 565. 31252173 10.1016/j.actbio.2019.06.036

[12] N. Lee , M. F. Horstemeyer , H. Rhee , B. Nabors , J. Liao , L. N. Williams , J. R. Soc. Interface 2014, 11, 20140274. 24812053 10.1098/rsif.2014.0274 PMC4032540

[13] C. Sanchez , H. Arribart , M. M. Giraud‐Guille , Nat. Mater. 2005, 4, 277. 15875305 10.1038/nmat1339

[14] N. A. Yaraghi , N. Guarín‐Zapata , L. K. Grunenfelder , E. Hintsala , S. Bhowmick , J. M. Hiller , M. Betts , E. L. Principe , J. Y. Jung , L. Sheppard , R. Wuhrer , J. McKittrick , P. D. Zavattieri , D. Kisailus , Adv. Mater. 2016, 28, 6835. 27238289 10.1002/adma.201600786

[15] Z. Jia , M. C. Fernandes , Z. Deng , T. Yang , Q. Zhang , A. Lethbridge , J. Yin , J. H. Lee , L. Han , J. C. Weaver , K. Bertoldi , J. Aizenberg , M. Kolle , P. Vukusic , L. Li , Proc. Natl. Acad. Sci. U. S. A. 2021, 118, e2101017118. 34140412 10.1073/pnas.2101017118 PMC8237622

[16] C. E. Hamm , R. Merkel , O. Springer , P. Jurkojc , C. Maiert , K. Prechtelt , V. Smetacek , Nature 2003, 421, 841. 12594512 10.1038/nature01416

[17] M. Hildebrand , S. J. L. Lerch , R. P. Shrestha , Mar. Sci. 2018, 5, 125.

[18] D. Losic , K. Short , J. G. Mitchell , R. Lal , N. H. Voelcker , Langmuir 2007, 23, 5014. 17397194 10.1021/la062666y

[19] A. M. Belcher , X. H. Wu , R. J. Christensen , P. K. Hansma , G. D. Stucky , D. E. Morse , Nature 1996, 381, 56.

[20] B. L. Smith , T. E. Schäffer , M. Vlani , J. B. Thompson , N. A. Frederick , J. Klndt , A. Belcher , G. D. Stucky , D. E. Morse , P. K. Hansma , Nature 1999, 399, 761.

[21] F. Marin , G. Luquet , C. R. ‐ Palevol 2004, 3, 469.

[22] F. Marin , G. Luquet , B. Marie , D. Medakovic , Curr. Top. Dev. Biol. 2007, 80, 209. 10.1016/S0070-2153(07)80006-8 17950376

[23] F. Marin , N. Le Roy , B. Marie , Front. Biosci. ‐ Sch. 2012, 4 S, 1099. 10.2741/s321 22202112

[24] Biomimetic Use of Food‐Waste Sources of Calcium Carbonate and Phosphate for Sustainable Materials—A Review, (Accessed: August, 2025), https://www.mdpi.com/1996‐1944/17/4/843. 10.3390/ma17040843 PMC10890559 38399094

[25] E. Beniash , C. A. Stifler , C. Y. Sun , G. S. Jung , Z. Qin , M. J. Buehler , P. U. P. A. Gilbert , Nat. Commun. 2019, 10, 4383. 31558712 10.1038/s41467-019-12185-7 PMC6763454

[26] E. A. Zimmermann , R. O. Ritchie , Adv. Healthcare Mater. 2015, 4, 1287.

[27] M. A. Meyers , P. Y. Chen , A. Y. M. Lin , Y. Seki , Prog. Mater. Sci. 2008, 53, 1.

[28] G. J. Vermeij , J. Zool. 1971, 163, 15.

[29] C. McDougall , B. M. Degnan , Wiley Interdiscip. Rev. Dev. Biol. 2018, 7, e313. 29470863 10.1002/wdev.313

[30] J. G. Carter , G. R. Clark , Notes Short Course Stud. Geol. 1985, 13, 50.

[31] Rigidity‐toughness coupling in architected composite materials for enhanced impact resistance ‐ ScienceDirect, (Accessed: August, 2025), https://www.sciencedirect.com/science/article/pii/S0020740324002339?casa_token=6IZDZjD7Vl8AAAAA:ztt3SO5pDsoVOigu‐LPlp3kz1ZC‐Cagy7a4PH55rJYjUaPsAvY9oeQwRXpjWZbfbCL72‐qE.

[32] D. Chateigner , C. Hedegaard , H. R. Wenk , J. Struct. Geol. 2000, 22, 1723.

[33] A. G. Checa , Front. Mar. Sci. 2018, 5, 353.

[34] G. J. Vermeij , Paleobiology 2002, 28, 41.

[35] F. Barthelat , J. E. Rim , H. D. Espinosa , Applied Scanning Probe Methods XIII, Springer Berlin, Heidelberg, Berlin Heidelberg 2008.

[36] C. Salinas , D. Kisailus , JOM 2013, 65, 473.

[37] C. L. Salinas , E. E. de Obaldia , C. Jeong , J. Hernandez , P. Zavattieri , D. Kisailus , J. Mech. Behav. Biomed. Mater. 2017, 76, 58. 28602753 10.1016/j.jmbbm.2017.05.033

[38] D. C. Rhoads , G. Pannella , Lethaia 1970, 3, 143.

[39] G. J. Vermeij , Evolution 1974, 28, 656. 28564826 10.1111/j.1558-5646.1974.tb00797.x

[40] G. Pannella , C. Macclintock , J. Paleontol. 1968, 42, 64.

[41] M. S. Clark , L. S. Peck , J. Arivalagan , T. Backeljau , S. Berland , J. C. R. Cardoso , C. Caurcel , G. Chapelle , M. De Noia , S. Dupont , K. Gharbi , J. I. Hoffman , K. S. Last , A. Marie , F. Melzner , K. Michalek , J. Morris , D. M. Power , K. Ramesh , T. Sanders , K. Sillanpää , V. A. Sleight , P. J. Stewart‐Sinclair , K. Sundell , L. Telesca , D. L. J. Vendrami , A. Ventura , T. A. Wilding , T. Yarra , E. M. Harper , Biol. Rev. 2020, 95, 1812. 32737956 10.1111/brv.12640

[42] M. A. Meyers , A. Y. M. Lin , P. Y. Chen , J. Muyco , J. Mech. Behav. Biomed. Mater. 2008, 1, 76. 19627773 10.1016/j.jmbbm.2007.03.001

[43] S. A. Watson , L. S. Peck , P. A. Tyler , P. C. Southgate , K. S. Tan , R. W. Day , S. A. Morley , Glob. Change Biol. 2012, 18, 3026. 10.1111/j.1365-2486.2012.02755.x 28741833

[44] F. Aguilera , C. McDougall , B. M. Degnan , D. Irwin , Mol. Biol. Evol. 2017, 34, 779. 28053006 10.1093/molbev/msw294 PMC5400390

[45] J. Peng , G. Jeffrey Snyder , Science 2019, 366, 690. 31699925 10.1126/science.aaz5704

[46] J. Ihli , A. S. Schenk , S. Rosenfeldt , K. Wakonig , M. Holler , G. Falini , L. Pasquini , E. Delacou , J. Buckman , T. S. Glen , T. Kress , E. H. R. Tsai , D. G. Reid , M. J. Duer , M. Cusack , F. Nudelman , Nat. Commun. 2021, 12, 5383. 34508091 10.1038/s41467-021-25613-4 PMC8433230

[47] C. Darwin , On the Origin of Species, Vol. 1859, Barns and Noble, NY, USA 2004.

[48] H. Nordsieck , Worldwide Door Snails (Clausiliidae), Recent and Fossil, ConchBooks, ConchBooks, Hackheim, Germany 2007.

[49] H. Nordsieck , Basteria 2002, 66, 85.

[50] S. Giokas , P. Pafilis , E. Valakos , J. Molluscan Stud. 2005, 71, 15.

[51] E. Gittenberger , D. S. J. Groenenberg , B. Kokshoorn , R. C. Preece , Nature 2006, 439, 409. 16437103 10.1038/439409a

[52] D. R. Uit De Weerd , D. S. J. Groenenberg , M. Schilthuizen , E. Gittenberger , Biol. J. Linn. Soc. 2006, 88, 155.

[53] V. Douris , S. Giokas , D. Thomaz , R. Lecanidou , G. C. Rodakis , Mol. Phylogenet. Evol. 2007, 44, 1224. 17320418 10.1016/j.ympev.2007.01.004

[54] K. Szybiak , M. Leśniewska , J. Molluscan Stud. 2008, 74, 183.

[55] H. Nordsieck , Arch. Molluskenkd. 2006, 135, 49.

[56] D. R. Uit de Weerd , E. Gittenberger , Mol. Phylogenet. Evol. 2013, 67, 201. 23357123 10.1016/j.ympev.2013.01.011

[57] A. Pohl , S. A. Herrera , D. Restrepo , R. Negishi , J. Y. Jung , C. Salinas , R. Wuhrer , T. Yoshino , J. McKittrick , A. Arakaki , M. Nemoto , P. Zavattieri , D. Kisailus , J. Mech. Behav. Biomed. Mater. 2020, 111, 103991. 32823075 10.1016/j.jmbbm.2020.103991

[58] W. Huang , D. Montroni , T. Wang , S. Murata , A. Arakaki , M. Nemoto , D. Kisailus , Acc. Chem. Res. 2022, 55, 1360. 35467343 10.1021/acs.accounts.2c00110

[59] X. W. Li , H. M. Ji , W. Yang , G. P. Zhang , D. L. Chen , J. Mech. Behav. Biomed. Mater. 2017, 74, 54. 28550764 10.1016/j.jmbbm.2017.05.022

[60] F. Du , S. Alghamdi , J. Yang , D. Huston , T. Tan , ACS Biomater. Sci. Eng. 2022, 9, 3843. 35959691 10.1021/acsbiomaterials.2c00080

[61] J. Sun , W. Wu , W. Xue , J. Tong , X. Liu , IET Nanobiotechnol. 2016, 10, 334. 27676383 10.1049/iet-nbt.2015.0082 PMC8676509

[62] L. K. Grunenfelder , E. E. De Obaldia , Q. Wang , D. Li , B. Weden , C. Salinas , R. Wuhrer , P. Zavattieri , D. Kisailus , Adv. Funct. Mater. 2014, 24, 6093.

[63] M. Yaso , T. Takaiwa , Y. Minagi , T. Kanaizumi , K. Kubota , T. Hayashi , S. Morito , T. Ohba , J. Alloys Compd. 2013, 577, S690.

[64] W. Huang , M. Shishehbor , N. Guarín‐Zapata , N. D. Kirchhofer , J. Li , L. Cruz , T. Wang , S. Bhowmick , D. Stauffer , P. Manimunda , K. N. Bozhilov , R. Caldwell , P. Zavattieri , D. Kisailus , Nat. Mater. 2020, 19, 1236. 32807923 10.1038/s41563-020-0768-7

[65] X. Zhang , Y. Yuan , Eng. Fract. Mech. 2023, 289, 109397.

[66] E. Gittenberger , T. D. Hamann , T. Asami , PLoS ONE 2012, 7, e34005. 22532825 10.1371/journal.pone.0034005 PMC3332057

