# Supplementary material for: Biogenic Design of the Flexible, Resilient, and Hard Mineral Protector in Door Snails
Source: Small Sci. 2025 Sep 28;5(12):e202500385. doi: 10.1002/smsc.202500385 (PMC12697901; doi:10.1002/smsc.202500385)
Supplement: Supplementary file 1 — Supplementary Material [file SMSC-5-e202500385-s001.zip › smsc202500385-sup-0001-SuppData-S1.pdf]

Biogenic design of the flexible, resilient, and hard mineral protector in door snails

Author Information

Yuri Kurihara[1], Taro Yoshimura[1,2,3], Ilian Häggmark[4], Rei Ueshima[3], Motoaki Hayama[1], Takuto Kishimoto[1], Nozomi Ono[1], Taige Hao[5], David Kisailus[5], Hidetoshi Takahashi[1], Hiroyuki Fujimoto[6], Kentaro Uesugi[7], Masato Hoshino[7], Yuya Oaki[1], Takenori Sasaki[2,3], Hiroaki Imai[1]*

1. School of Integrated Design Engineering, Graduate School of Science and Technology, Keio University, Kanagawa, Japan
   E-mail: hiroaki@applc.keio.ac.jp

2. The University Museum, The University of Tokyo, Tokyo, Japan

3. Graduate School of Science, The University of Tokyo, Tokyo, Japan

4. Department of Applied Physics, KTH Royal Institute of Technology, Stockholm, Sweden

5. Department of Materials Science and Engineering, University of California, Irvine, California, USA

6. Shimadzu Corporation, Kyoto, Japan

7. Japan Synchrotron Radiation Research Institute (JASRI/SPring-8), Hyogo, Japan
   Hyogo, 679-5198, Japan

Keywords
biomimetic materials, hierarchical architectures, mollusk shells, snail evolution, structure–function relationships

Abstract
Organism design incorporates diverse materials with varying properties, such as hard skeletons of biogenic minerals and soft organic skins. However, achieving a balance of flexibility, resilience, and hardness remains a challenge even for organisms. Door snails have a calcareous door (clausilium) that covers the aperture. The clausilium combines hardness for defense and flexibility for opening and closing. Here we focus on the biogenic design of a clausilium stalk as a unique architecture balancing several properties. This study investigates the stalk, a twisted ribbon with high flexibility and resilience, identified as a synapomorphic structure in 22 Clausiliidae species across 7 subfamilies and 17 tribes. Internal observations reveal a double-layered structure: a hard, dense envelope with aragonite rods arranged in the b axis and a flexible, low-density core with randomly packed aragonite nanoparticles and organic matter. The anisotropic hierarchical design found in nature will surely be useful in the development of artificial materials that combine flexibility, resilience, and hardness.

Funding

Japan Society for the Promotion of Science KAKENHI grant JP21H01627 (HI)

Japan Society for the Promotion of Science KAKENHI grant 23KJ0818 (TY)

the Early Career Research Grant 2020 (TY)

The Malacological Society of London and J Frances Allen Institute of Malacology Student Research Award 2021 (TY)

Conflict of interest

Authors declare no conflict of interest.

Data Availability Statement

The authors declare that the data supporting the findings of this study are available within the paper and its Supplementary Information file. Raw data files are available from the corresponding author upon reasonable request.

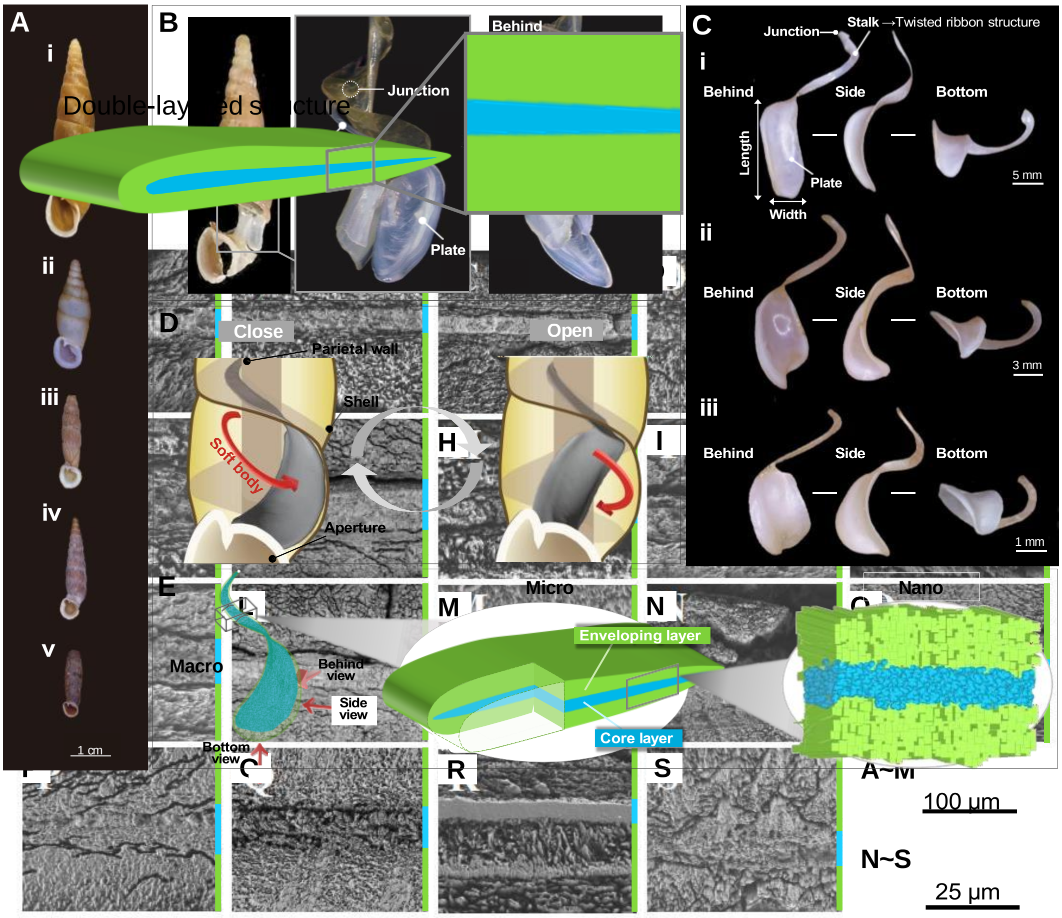

Figure S1

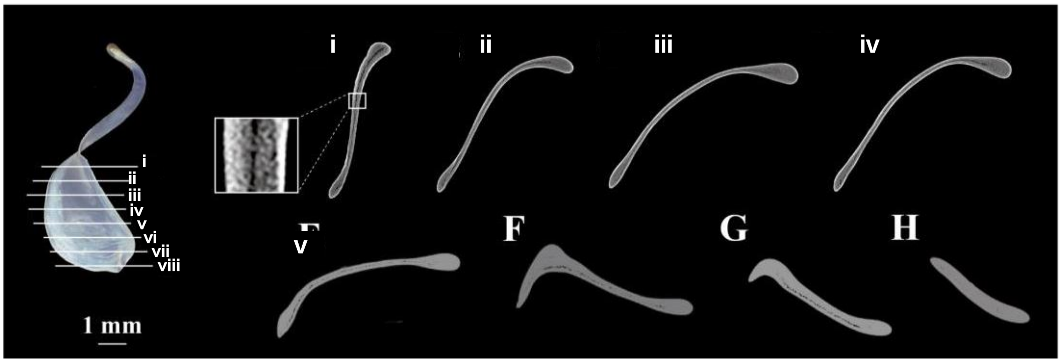

Figure S2

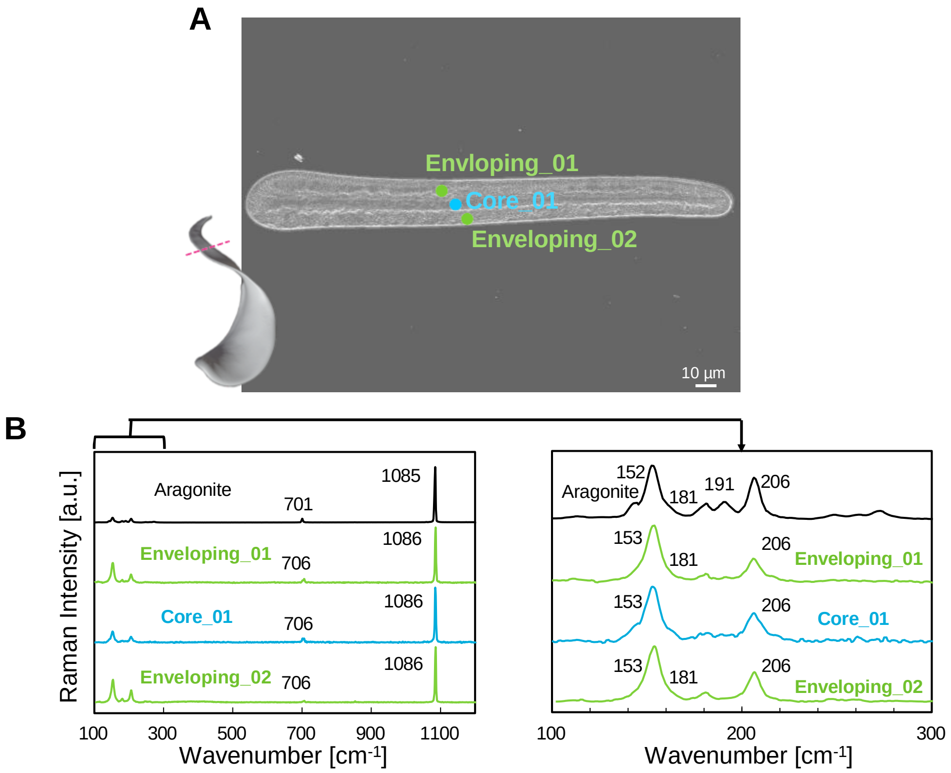

Figure S3

A

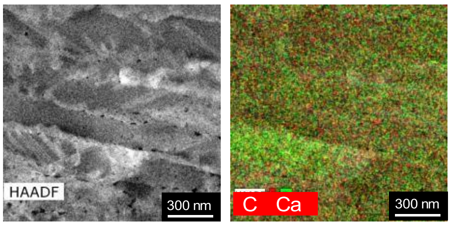

Element	wt. %	atm %
C	14.66	24.72
N	1.28	1.85
O	40.72	51.53
Ca	43.34	21.90

B

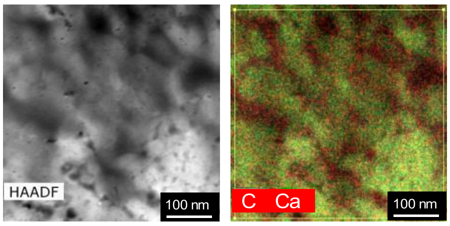

Element	wt. %	atm %
C	39.75	53.45
N	4.45	5.13
O	31.24	31.53
Ca	24.56	9.90

C

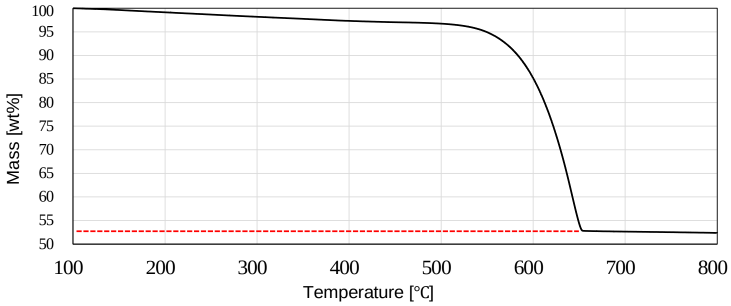

Figure S4

A

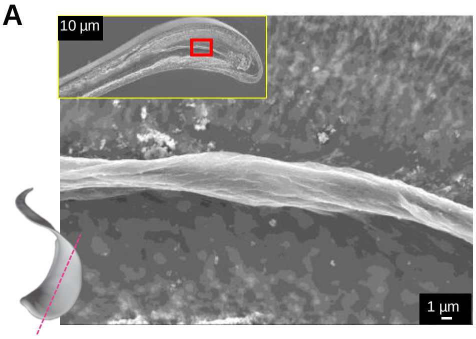

B

Element	wt. %	atm %
C	22.98	35.20
N	5.58	7.33
O	37.61	43.27
Ca	28.34	13.01

Figure S5

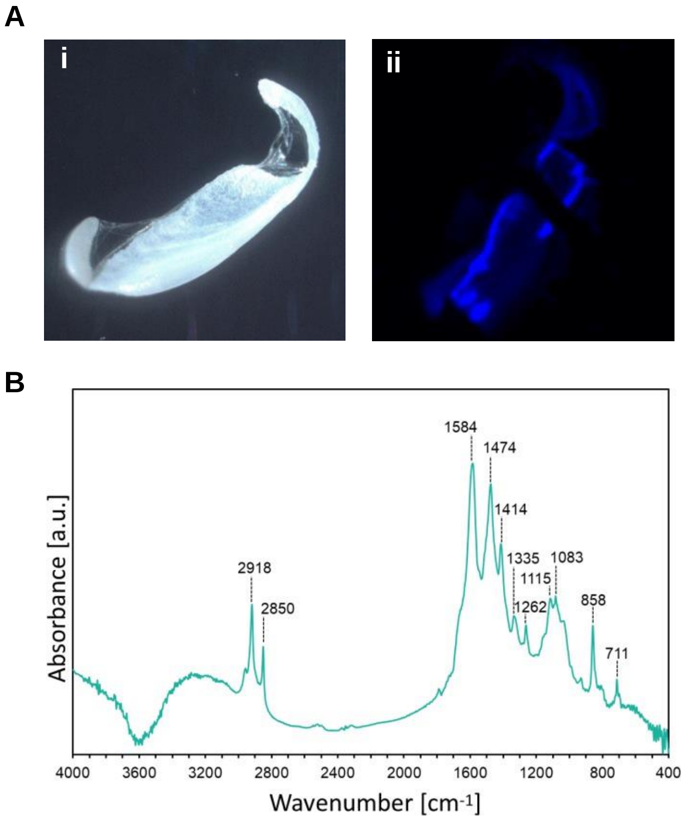

Figure S6

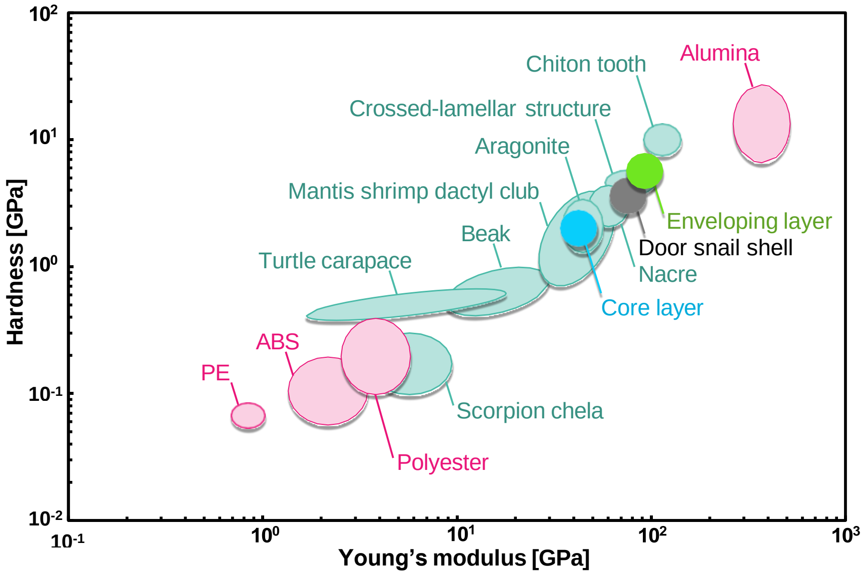

Figure S7

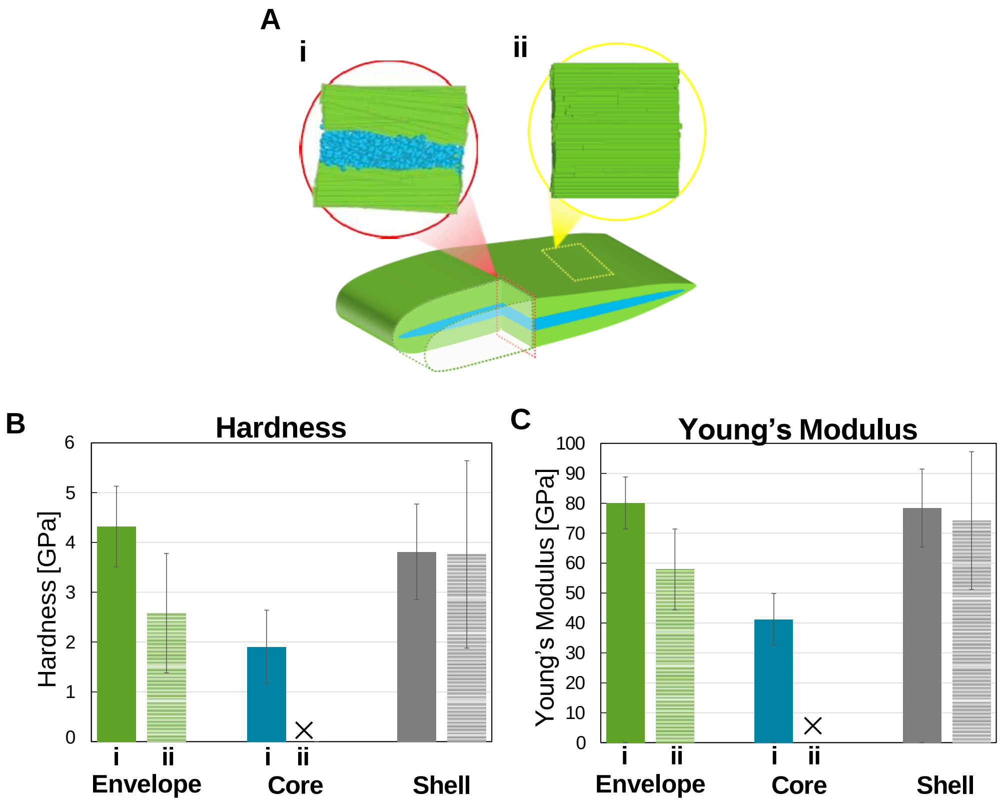

Figure S8

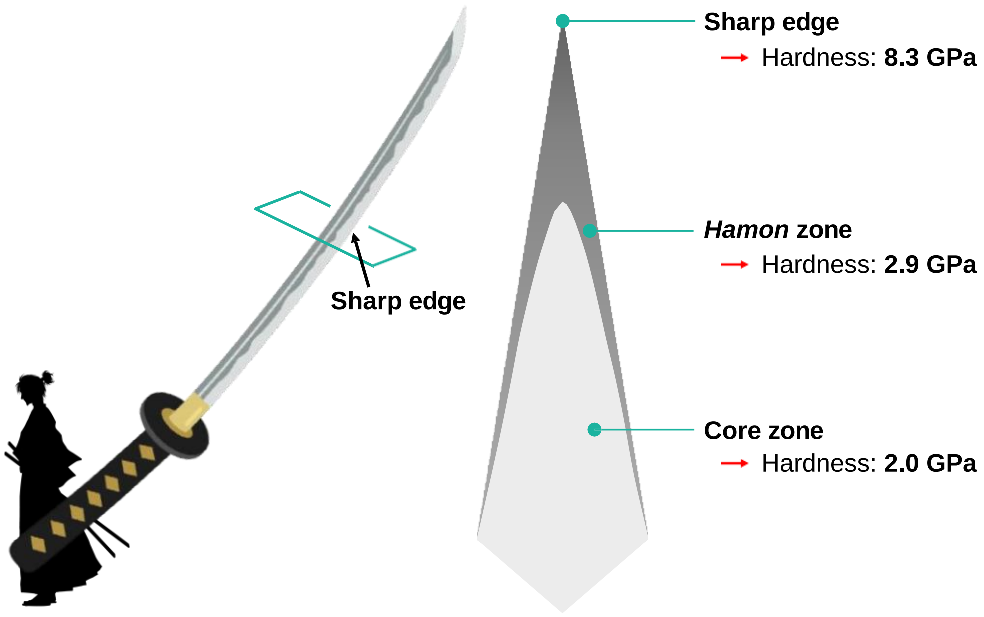

Figure S9

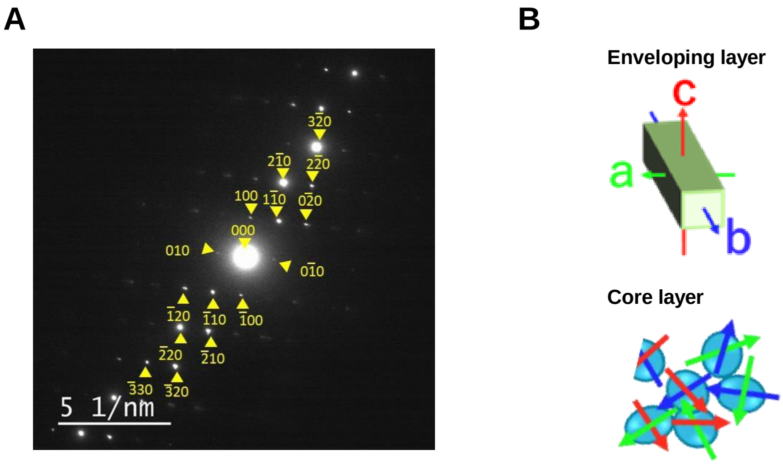

Figure S10

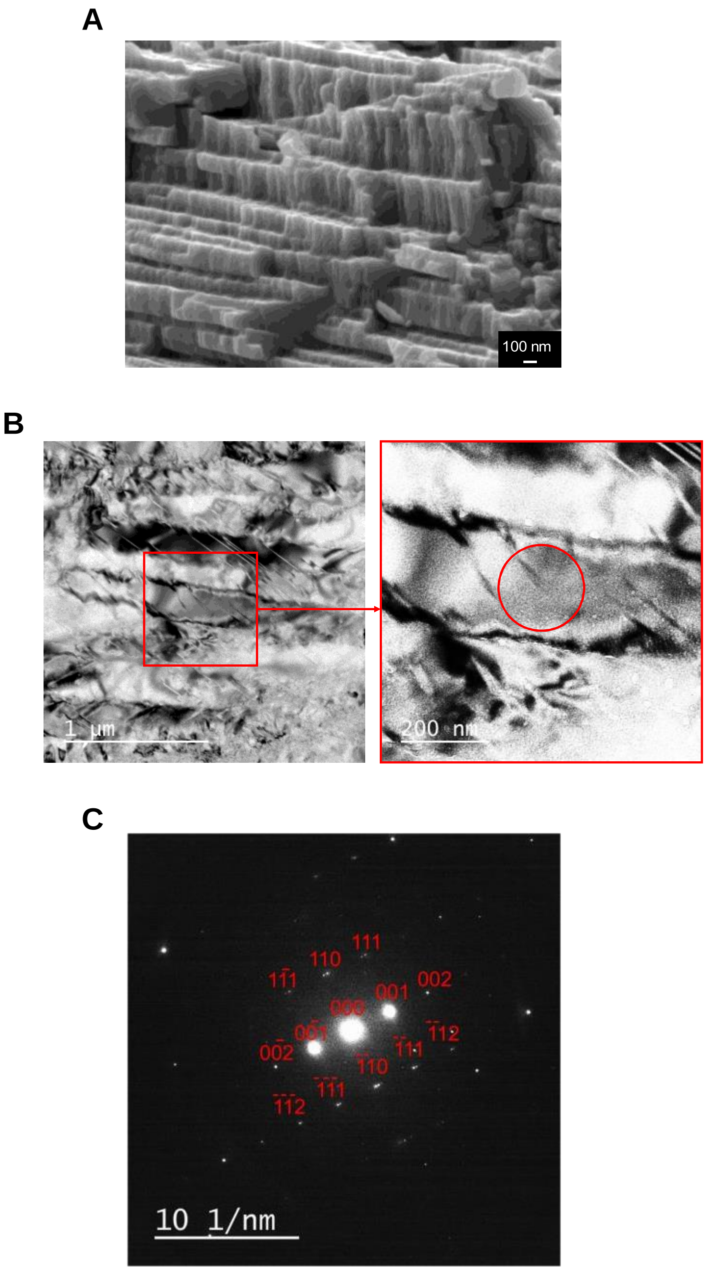

Figure S11

Table S1

Subfamily (Tribe)	Species	No.	Locality
Alopiinae (Alopiini)	*Alopia livida*	UMUT-TY-GHT096	Ordincusa-Valea, Mt. Bihar, Romania
Alopiinae (Cochlodinini)	*Cochlodina laminata*	UMUT-TY-GHT096-2	Kuehkopf, Hessen, Germany
Alopiinae (Delimini)	*Charpentieria stenzii*	UMUT-TY-GHT096-3	Lod Pod Mangartom, Juliai Alps, Slovenia
Alopiinae (Medorini)	*Medora almissana*	UMUT-TY-GHT096-4-2	Omisicetina Valley, Hungary
Clausiliinae (Acrotomini)	*Roseniella sobrievskii*	UMUT-TY-GHT097	Ardanuc, Artvin Vilayet, Turkey
Clausiliinae (Baleini)	*Laciniaria plicata*	UMUT-TY-GHT097-2	Loutro Arides, Nomos Pela, Greece
Clausiliinae (Boettgeriini)	*Boettgeria crispa*	UMUT-TY-GHT097-3	Ribeiro Frio, Madeira Island, Portugal
Clausiliinae (Clausiliini)	*Clausilia dubia*	UMUT-TY-GHT097-4	Monastery of Ste Odile, Alsace, France
Clausiliinae (Filosini)	*Idyla castalia crenilabris*	UMUT-TY-GHT097-6	Lithohoro, Makedonia, Greece
Clausiliinae (Mentissoideini)	*Elia moesta*	UMUT-TY-GHT097-8	Azon Island, Israel
Clausiliinae (Olympicolini)	*Olympicola olympica*	UMUT-TY-GHT097-9	Bursa Vilayet, Turkey
Clausiliinae (Strigileuxinini)	*Strigileuxina concavelamellata*	UMUT-TY-GHT097-10	5 km South Hamsiköy, Trabzon Village, Turkey
Garnieriinae (Garnieriini)	*Garnieria mouboti yunnancola*	UMUT-TY-GHT098-1	YunNan Province
Garnieriinae (Tropidaucheniini)	*Tropidauchenia mirifica*	UMUT-TY-GHT098-2-2	Guigang City, Guangxi Province, China
Laminiferinae (Bofilliellini)	*Bofilliella subarcuata*	UMUT-TY-GHT99-1	La Vall del Bac, Girona Pr., Catalonia, Spain
Neniinae	*Nenia tridens*	UMUT-TY-GHT100	Luquillo Rain Forest, USA
Peruiniinae	*Brevinenia richardsi*	UMUT-TY-GHT101-1	65 km from Balsas to Leymebamba, Dept. Amazonas, North Peru.
	Parabalea balnearum	UMUT-TY-GHT101-2	3700m, Cusco Parapucara, Peru
Phaedusinae (Phaedusini)	*Megalophaedusa martensi*	UMUT-TY-GHT102-1-5	Tosa, Kohchi, Japan
	Stereophaedusa japonica japonica	UMUT-TY-GHT102-1-18	Hiyoshi, Yokohama, Kanagawa, Japan
	Tauphaedusa tau	UMUT-TY-GHT102-1-28	Hiyoshi, Yokohama, Kanagawa, Japan
Phaedusinae (Serrulinini)	*Serrulina sieversi*	UMUT-TY-GHT102-3	Abbasabad, Iran
